# Effects of Strength Training on BDNF in Healthy Young Adults

**DOI:** 10.3390/ijerph192113795

**Published:** 2022-10-24

**Authors:** Miroslaw Babiarz, Radoslaw Laskowski, Tomasz Grzywacz

**Affiliations:** 1 Department of Physiology, Gdansk University of Physical Education and Sport, 80-336 Gdansk, Poland; 2 Department of Sport, Institute of Physical Education, Kazimierz Wielki University in Bydgoszcz, 85-604 Bydgoszcz, Poland

**Keywords:** strength training, cognitive function, resistance training, brain-derived neurotrophic factor (BDNF)

## Abstract

The physical improvements from strength and resistance training have been known for decades, but the cognitive benefits of this type of activity are not as well-known. The aim of this review article is to provide a summary of studies presenting the effects of strength and resistance training on BDNF in healthy young adults. A systematic search of various electronic databases (PubMed, Web of Science, Science Direct and Google Scholar) was conducted in September 2022. Studies that examined associations between strength training and BDNF in healthy young adults aged 18–30 years were included. The final sample included 10 studies published between 2009 and 2020. The results of this literature review are inconclusive. Based on the results of the 10 studies, there is no clear answer as to whether strength training has positive effects on BDNF in healthy young adults.

## 1. Introduction

Strength training is a staple for physical [1] and mental health [2]. The benefits are not only stronger bones [3], ligaments [4], tendons and muscle tissues [5] but also a more capable mind [6]. In research databases, most studies that link strength training to cognition are primarily related to the elderly [7] and the military [8]. Other studies focusing on cognition and physical activity use endurance-based protocols, and their predictor of exercise intensity is often VO2 max [9,10,11,12].

Cognitive function refers to mental processes involved in the acquisition of knowledge and understanding through thought and experience [13]. Cognitive function is critical for all voluntary actions, including academic achievement, occupational success, and functional independence [14]. The BDNF protein is critical for acute cognitive performance in the short term and for brain morphology adaptations (e.g., plasticity) in the long term [15]. Brain Derived Neurotrophic Factor (BDNF) is a highly conserved neurotrophic protein of the nerve-growth factor family. Its main function is the regulation of synapses, with structural and functional effects in many brain regions. There, it promotes neuron survival, neuroplasticity, neurite growth (the process by which developing neurons form new processes) and synaptogenesis (the formation of synapses between neurons in the nervous system) [16,17]. BDNF also has a major impact on learning and memory and has been identified as a key component of the hypothalamic signaling pathway. This explains why BDNF controls body weight, decreases food intake, lowers blood glucose levels and controls energy homeostasis [18,19]. The effects of BDNF isoforms on structural changes in the brain are not applicable to studies of acute stress, as they are more likely to be chronic effects (which may be relevant to studies of long-term stress). Nevertheless, BDNF isoforms have also been observed to affect neuronal activity by being associated with cellular models of memory (i.e., long-term potentiation and long-term depression) [15]. Approximately 70–80% of circulating plasma BDNF, both during exercise and at rest, originates from the brain. It appears that the brain is a mainstay for increasing BDNF plasma during exercise and recovery in healthy individuals [18].

**Figure 1:**
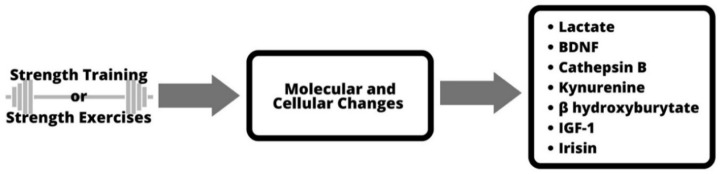
Molecular and cellular mechanisms of strength training.

Additional studies are needed to expand our knowledge of the relationship between strength-training- and/or resistance-training-induced expression of (serum) BDNF in humans and its relationship to functional and structural brain changes and cognitive performance [2]. At the molecular and cellular level, Figure 1 shows the proposed process/mechanism. The changes in blood lactate concentration and serum level of insulin-like growth factor 1 are some of the factors that cause a pronounced release of BDNF [2]. The figure also shows other mechanisms, such as cathepsin B and kynurenine, by which active skeletal muscle can affect the brain [20].

Because BDNF protein controls the survival and maintenance of nerve cells (neurons), it also regulates synaptic plasticity, memory and learning ability. For this reason, the possible changes in BDNF levels after resistance training are very accessible. BDNF protein is critical for both short-term and long-term (brain plasticity) cognitive performance and adaptation [21]. BDNF signaling pathways are largely regulated by tropomyosin-related receptor kinase B (TrkB) [22]. TrkB influences neuronal dendritic afforestation, spinogenesis, dendritic growth and spine morphogenesis [23]. The exact process and mechanism by which exercise increases BDNF, leading to changes in neuroplasticity, is not yet fully understood [23]. It is speculated that it is because of BDNF abundance in brain regions associated with plasticity as well as activity-dependent regulation of expression and secretion [24].

The following review focuses on available research on the BDNF activation process exclusively after resistance training in healthy young adults. Only studies that specifically used strength training as a variable were considered, and studies on aerobic and endurance training were excluded, as these have been previously reviewed [9,10,11,12]. This topic is critically important because overall cognitive improvements are crucial at any age, and the sooner we find a way to improve our cognitive abilities naturally, the longer we will stay young.

## 2. Materials and Methods

**Figure 2:**
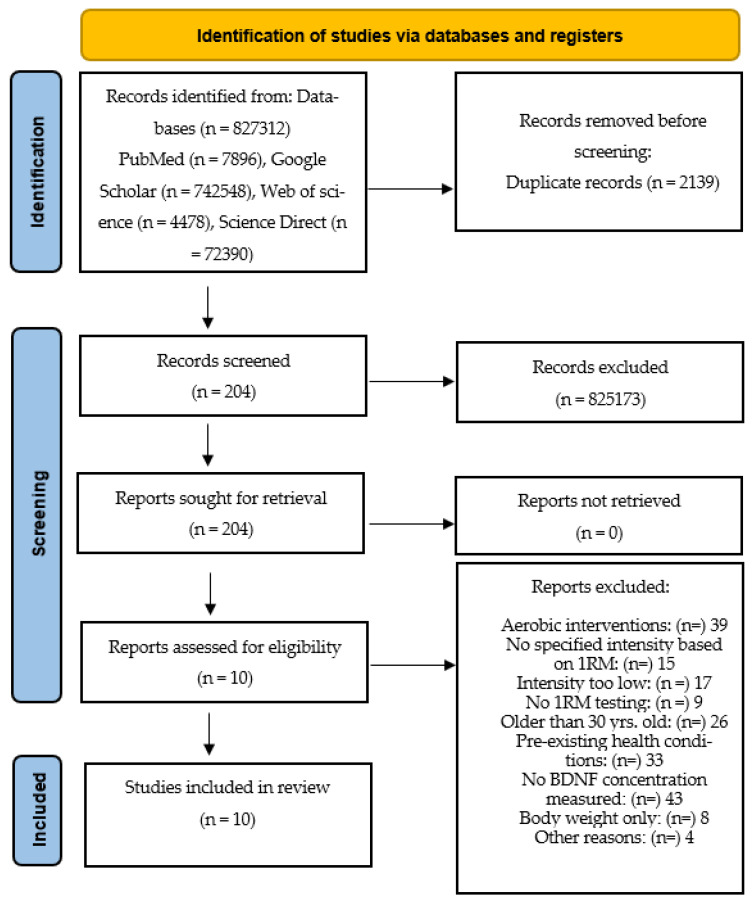
Flowchart providing information on the search, screening and selection processes that led to the identification of relevant articles included in this systematic review.

This study reviewed the literature on BDNF and strength training in healthy young adults. A systematic search of various electronic databases (PubMed, Web of Science, Science Direct and Google Scholar) was conducted in September 2022. Studies that examined associations between strength training and BDNF in young adults aged 18–30 years were included. The detailed flowchart of the literature search and study selection is shown in Figure 2.

Identification of studies: the selected studies that met the criteria were exclusively strength training studies. Endurance studies were excluded. The selected strength training studies included only those that had a specific training intensity. Training intensity is measured based on the percentage of one repetition maximum (1RM). For example, an intensity of 80% means that subjects performed all exercises at that intensity. The population for the review included only young adults, some of whom were athletes. There was a total of 10 studies with 167 participants published between 2009 and 2020 that met the inclusion criteria. The following terms were used as search strings:

“BDNF strength training” OR “BDNF resistance training” OR “BDNF resistance exercise” OR “BDNF strength exercise” OR “BDNF weightlifting” OR “BDNF physical training” OR “BDNF strength and conditioning” OR “BDNF sports performance” OR “BDNF fitness” OR “BDNF muscular strength” OR “BDNF hypertrophy” OR “BDNF functional training”

AND

“brain-derived neurotrophic factor strength training” OR “brain-derived neurotrophic factor resistance training” OR “brain-derived neurotrophic factor resistance exercise” OR “brain-derived neurotrophic factor strength exercise” OR “brain-derived neurotrophic factor weightlifting” OR “brain-derived neurotrophic factor physical training” OR “brain-derived neurotrophic factor strength and conditioning” OR “brain-derived neurotrophic factor sports performance” OR “brain-derived neurotrophic factor fitness” OR “brain-derived neurotrophic factor muscular strength” OR “brain-derived neurotrophic factor hypertrophy” OR “brain-derived neurotrophic factor functional training”

Inclusion/Exclusion Criteria:

Inclusion:

1. intensity: specified percentage of 1 rep maximum (% of 1RM). Only studies whose training was based on a specified percentage (intensity) and that tested maximal weight (for the exercises used in the study) prior to starting the exercise protocol were included.
2. young and healthy subjects only (women and men aged 18–30 years).
3. health: no pre-existing medical conditions.
4. Studies that performed strength training with machines or free weights were included.

Exclusion:

5. small sample size (n < 10)
6. use of cigarettes, alcohol, drugs.
7. Pre-existing health conditions such as:
8. Any type of heart disease or nervous system disease
9. Any type of disability
10. Any type of bone or muscle degenerative disease
11. Any type of other disease that would prevent the exercise and performance of strength training exercises with the required technique and intensity/volume protocols
12. Endurance or body weight exercise studies were excluded.

The above criteria were chosen to distinguish this systematic review from other resistance training reviews written to date [25,26,27]. This increases its value by narrowing the criteria to a specific group of young, healthy adults performing strength training exercises with specific training parameters. The use of the percentage of 1 repetition maximum and the performance of resistance exercises exclusively with machines or free weights also increases validity and is aimed mostly at strength training enthusiasts (researchers, athletes and/or students).

## 3. Discussion

### 3.1. Positive BDNF Response

There were four studies [28,29,30,31] that found positive BDNF response to strength training. In contrast, there are six other studies [16,32,33,34,35,36] that found no difference between BDNF levels before and after training. Table 1 illustrates all these differences between the studies in detail. Martson et al. [28] examined the effect of acute strength training to fatigue on serum BDNF levels both immediately after and 30 min after exercise. Sixteen subjects aged 22 to 26 years, who had not previously undergone resistance training, performed either a hypertrophy or strength training protocol. Participants completed a total of four sessions, including two familiarization sessions and two experimental sessions. In the familiarization sessions, participants learned seven strength training exercises (bench press, pull down, leg press, leg extension, seated rowing, military press and dumbbell curl). Maximum strength for each of the seven exercises was measured at a maximum of five repetitions (5RM) and at a maximum of ten repetitions (10RM). In the following two sessions, subjects completed either a strength-based or a hypertrophy-based resistance training protocol in a randomized order. Venous blood samples were collected before the start of the warm-up protocol, immediately after the completion the training session and then 30 min after the session. An interaction was observed between the conditioning with hypertrophy protocol; it resulted in higher serum BDNF levels compared to the strength protocol. The strength protocol did not result in changes in BDNF levels. The hypertrophy protocol resulted in a 13% increase in serum BDNF levels compared with baseline levels. The study concluded that the use of a hypertrophy exercise protocol performed to failure provides the necessary stimulus to increase peripheral serum BDNF. The authors used a relatively small sample of volunteers who had no experience with resistance training. For this reason, we should question the validity of the five and ten maximum repetition tests and of the training itself. The participants used the tested weight later in the workout, and anyone who has experience with strength training knows that it is impossible to repeat the same maximum weight tested with only 60 s rest for three sets in 7 separate exercises, as was the case for the hypertrophy group. In addition, the authors explain that the measurement of BDNF levels in the blood at the end of the exercise session may not be representative of maximal BDNF expression. This is because larger muscle groups were exercised at the beginning of the training session, which could possibly lead to a greater release of BDNF due to the greater blood flow before the actual measurements at the end of the training session. Yarrow et al. [31] found that BDNF expression appears to be relatively transient, decreasing to baseline or below within 30 min of exercise.

Lira et al. [29] used whole-body and split-body resistance training routines to examine the immunometabolic response to brain-derived neurotrophic factor. The hypothesis was that whole-body resistance training would produce a greater increase in serum BDNF than split-body resistance training would. The sample consisted of twelve men (age = 25.3 ± 5.9 years) whose experience with resistance training protocols was purely recreational. They conducted three randomized trials with 18 training sets: upper-body (UB), lower-body (LB) and full-body (FB). Serum BDNF levels were measured at rest, immediately after exercise, 1 h after and 2 h later during recovery. The result showed the largest effect size for the lower-body group (1.4), which was almost twice that of the whole-body group (0.75) and more than four times that of the upper-body group (0.33). However, no significant differences were found between conditions. It is suggested that the volume of work performed by larger muscles (in this case the lower body) has a larger influence on BDNF than total volume does.

To distinguish between volume and intensity and to determine which outcome is critical to the BDNF response, the third study is helpful. Church et al. compared high-intensity to high-volume resistance training in terms of BDNF response to exercise [30]. Twenty men with at least two years of strength training experience underwent a 9-week training protocol. The protocol included 2 weeks of preparation and 7 weeks of training. Groups were divided into either a high-intensity group or a high-volume group of 10 men each. Both groups trained four times per week, performing mainly multi-joint movements with free weights, such as squats, deadlifts, bench press, shoulder press, chin up, leg press, triceps extensions, etc. A series of tests were also performed, such as anthropometric assessment (height, weight and body composition) and a strength test (1RM in barbell squat and barbell bench press). Blood samples were also collected at four different time points, and nutrient intake and diet were analyzed. The results indicated that BDNF concentrations were significantly increased in both the high-intensity and high-volume groups. The results showed increased circulating BDNF concentrations but no change in resting concentrations after 7 weeks of training protocol. This study is very similar to that of Martson et al. [28] in that the same rest periods, a very similar repetition and set scheme and the use of multi-joint, free-weight exercises were used. The only difference was that the authors measured BDNF levels 60 min post-exercise in addition to immediately post- and 30 min post-exercise levels. This was also the longest study with positive results after 9 weeks and a total of 40 training sessions with 20 experienced, resistance-trained subjects.

Positive BDNF changes following resistance training were previously demonstrated in 2010 by Yarrow et al., when they found that training enhanced resistance-training-induced increases in circulating BDNF [31]. The group consisted of 20 college males who underwent traditional or eccentric enhanced resistance training for 5 weeks. Both groups completed three resistance training sessions per week. Blood was collected for analysis at rest and 1, 30 and 60 min after training. The results presented showed that BDNF levels were not altered at rest. After exercise, serum BDNF levels increased by 32% but decreased to 41% below resting levels. That occurred 60 min after training, already during a recovery phase. This study showed that resistance training causes a strong, but transient increase in circulating BDNF.

### 3.2. Negative BDNF Response

Lodo et al. presented the most recent study showing a negative response of BDNF to resistance training [32]. They found that resistance training had no effect on the response of neurotrophic factors in schemes of equal volume. The two-week study consisted of two resistance training sessions with one week of rest between the sessions with a total of 30 participants. Before starting the protocol, the 1RM test was performed for the bench press and squat, and blood was drawn 10 min before and 10 min after each training session to analyze serum BDNF levels. The results suggest that the intensity of resistance training is not a significant factor on the neurotrophic factor response when the total load lifted is equated in the range of submaximal repetitions. Only a small effect size was found for BDNF before and after training. The limitation of this study is certainly the same rest period of 120 s for both groups with ten and five repetitions. We might suspect that 120 s is too long, especially for the ten-repetition group, since two previous studies, Martson et al. [28] and Church et al. [30], which also used ten repetitions, found positive results in BDNF expression, both using 60 s of rest. In addition, the ten repetitions were performed at an intensity of only 35% and the five repetitions were performed at 70%, which is not even close to the 70% for ten repetitions and the 85–90% for five repetitions that both Martson et al. [28] and Church et al. [30] identified as triggering a positive BDNF response.

In the other study, Quiles et al. measured the impact of the configuration of the resistance training program on the circulating BDNF response [33]. Fifteen men with at least 2 years of resistance training experience (age: 23 ± 3 years, body weight: 84.4 ± 12.3 kg) participated. The training lasted for 6 weeks, and it consisted of 3×/week protocol and two different groups. The results showed no significant interactions or main effects for BDNF. There was a slight between-group effect (0.57) for the lower-repetition group in week 6. The hypothesis is that there is a minimum intensity and volume of resistance training required to produce an acute increase in BDNF. The average weekly intensity was 80% in the low-repetition group and only 65% in the high-repetition group. Total tonnage was certainly higher in the lower-repetition group. The results suggest that greater proximity to one repetition maximum may be required to elicit a BDNF response.

Vega et al. measured the effects of resistance training on serum levels of growth factors in humans before and after training [34]. Eleven adults performed two trials of isokinetic work with three repetitions maximum effort for the knee extension exercise. During a familiarization session, subjects alternated between concentric and eccentric work at a velocity of 60 degrees/s. The average torque angles from the three repetitions performed during the trials were later used as benchmarks for the test sessions. For the trials, the intensity was based on the results of the familiarization session and the averaged personal maximal effort curve. None of the trials showed significant difference in BDNF value compared to the value before training and only a slight increase in value during training compared to the value after training.

Another study also used two individual, separate training sessions. Correia et al. measured the acute strength training and the effect of small or large muscle mass on plasma BDNF levels [35]. Sixteen young adult men (age: 26.4 ± 3.7 years.), who had not previously exercised, performed strength measurements for knee and elbow flexors and extensors for both limbs, separately. Using a Cybex 6000 isokinetic dynamometer, knee muscles were tested on test day 1 and the elbow muscles on test day 2. The knee muscles were tested in a seated position with the hip flexed to 85 degrees and the range of motion set to 110 degrees (full flexion at 0 degrees). One week later, the elbow muscles were tested in the supine position with the range of motion set to 130 degrees (full extension 0 degrees). The shoulder was abducted to 45 degrees in the frontal plane with the forearm held neutrally. The same parameters were used for the knee and elbow muscles. This was the first study in this review that reported the time under tension (TUT) for each set performed. Thus, we know that the set of 10 repetitions lasted approximately 30 s for the knee muscles and 43 s for the elbow muscles. Blood sampling was performed at 1 min and 30 min following the exercise protocol. The results showed no significant results for plasma concentrations of BDNF in either the upper- or lower-body protocols. Although this was one of only two studies in this review that did not report training intensity based on one repetition maximum, it was included because of the quality of the procedures and tests. On the other hand, the rest periods between performed sets seem to be very short (40 s), especially since the minimum rest period at which a positive BDNF response to resistance training was detected was 60 s [28,30]. Another reason that may have a negative impact on the results is that neither multi-joint nor free-weight training was used in the protocol, nor did the authors specify an intensity based on a maximal strength test. The authors agree that the level of exercise is important for the change in BDNF, that the response varies depending on the intensity and type of exercise and that longer studies are needed to confirm the results.

Goekint et al. investigated the acute effect of strength training on BDNF during a 10-week program [16]. Fifteen previously untrained subjects took part in the study, and in addition there were eight control subjects who had done no training. Blood samples were taken at various times during the study (before the start, after the 6th session and after the last, 30th session). The results showed no effect on BDNF, which was measured in serum samples.

In the other study, Schiffer et al. measured the effects of strength training on BDNF during a 12-week intervention [36]. The 27 subjects were all healthy sports students (age: 22.2 ± 1.8 years) who had played team sports such as basketball, handball or football at some point but had no experience with strength training. Physical tests were performed before and after completion of the study to assess the strength level of each individual. Participants measured knee extensor muscle strength isometrically (at 120 degrees of knee flexion) and dynamically (at 90 degrees of knee flexion). The intervention lasted 12 weeks and consisted of whole-body training on exercise machines three times per week. Various exercises were performed such as: leg extension, leg curls, bench press, pull-down, leg press, seated rows, crunches, hyperextensions and lateral raises. Blood samples were collected to analyze plasma BDNF concentration before and after the 12-week intervention. The results showed that this study did not produce sufficient adaptations to basal plasma BDNF concentrations. Notably, BDNF levels were partially associated with reversed responses in all intervention groups. Thirteen of twenty-seven subjects had pre–post differences of more than 50 up to 230 pg-ml^−1^. Consequently, statistical analysis revealed no pre–post difference for BDNF other than high standard deviations. Although this was well-constructed, with longer intervention and with a large sample size, some limitations should be noted. First, no specific rest periods were used, which is important because of previous studies with similar modalities that differed only in rest periods and led to conflicting results [28,30,35]. In addition, only knee extension was tested in the strength group before and after the intervention, but many other exercises that had not been previously tested were used during the 12 weeks. The authors specified a certain intensity (70–80%) for the other exercises as well. This would not be possible if these exercises had not been tested for maximal strength.

## 4. Limitations

None of the 10 studies reported tempo when in relation to exercises. The four-digit tempo is used to assess the time it takes to perform each part of the exercise (e.g., squat). The eccentric part, the pause between the eccentric and the concentric part, the concentric part and the pause between the concentric and the eccentric parts are all symbolized by a single-digit number and in seconds. For example, tempo 4010 for a squat means: 4 s eccentric, 0 s pause (no pause), 1 s concentric and 0 s (no pause) on top. Tempo is used to emphasize a part of the lift, to increase or decrease the seconds it takes to perform each part of the lift or to pause at the bottom or top of the lift (even in the middle of the concentric or eccentric movement). Tempo parameters are individualized based on the demands of the training cycle. None of the 10 studies used or mentioned the use of tempo. Many studies show that tempo is important and that different tempos should be considered when designing resistance training programs for strength or hypertrophy [37,38]. Table 2 shows that tempo can also be used to determine the time under tension, which is used to see which strength type is developed during the performance of a set of the exercise.

There are a number of studies that show that time under tension is a key component in achieving a specific training adaptation. For example, for the development of relative strength, the total time under tension per set should not exceed 20 s [40,41,42,43]. The time under tension is closely correlated with the number of repetitions a person performs at a given tempo. For this reason, training effect is also determined by the number of repetitions performed for a given time under tension [43,44,45,46,47,48,49,50].

Another limitation is that specific rest periods have only been mentioned in five out of the ten selected studies. Rest time determines whether creatine phosphate (CrP) is recovered before the next set, which research suggests can take as long as 15 min [23]. Rest times are relative and depend on many factors, such as the extent of CrP depletion (based on the intensity of 1RM), the characteristics of the motor unit and muscle fibre type, aerobic and/or anaerobic fitness level, etc. The efficiency of recovery can also be improved by diet and creatine and phosphagen supplementation [51]. In general, the phosphagen system recovers to about 70% within 30 s and to 100% within 3 to 5 min in most cases [52]. The only studies that used specific (correct) rest periods found a positive BDNF response to a resistance training protocol [28,30,31]. Several other studies demonstrate that the length of the rest interval depends on the training goal and that a correct rest interval must be used to achieve a specific training goal [53,54,55,56,57,58,59].

Finally, intensity often did not match the 1RM continuum. This means that the intensity could be too low to perform a required number of repetitions. In just one example, a training protocol was performed with 10 repetitions at 35% [32]. In Table 3, we can clearly see that a person can perform 10 repetitions at much higher intensity. In order for the 10 repetitions to be performed at the correct intensity, the latter would need to be set at 74% and not at 35% [47,48,60]. Intensity (weight) should be adjusted and based on the number of repetitions a person can perform, which gives us an idea of what intensity they are training at. Relative strength, for example, is best trained between one and five repetitions, and this gives us a specific intensity percentage at which a person is training if they can do less than five repetitions [47,48,61,62,63]. If the intensity is set too low for performing the required number of repetitions, the results are not entirely valid. This is because the participants did not train at their highest available strength and energy but limited themselves by not going all the way to the last possible repetition at a given intensity. For example, the best intensity for developing maximal strength is between 70% (twelve repetitions) and 90% (three repetitions) [47,64,65,66,67,68,69,70,71].

The 1RM continuum provides a general estimate of the repetition ranges a person can perform at different percentages. It may vary from person to person and from muscle group to muscle group. For example, the number of repetitions one can perform at the same percentage for upper-body exercises for the triceps muscle is much lower than the number of repetitions one can perform for lower-body exercises such as the leg press [40,56,57,58,72,73,74,75,76]. Moreover, the number of repetitions a person can perform at a given percentage of 1RM is based on training age. Training age refers to the number of years a person has participated in and committed to a serious and consistent strength training protocol. Two years equals two years of training age, four years equals four years of training age, and so on. For example, the number of repetitions a beginner can perform at 75% of the 1RM is typically 20. With a training age of 3 years, he or she may be able to perform 10 repetitions at 75% of 1RM, and as training age increases, the number of repetitions performed at the same percentage decreases even further. [77,78,79,80,81].

## 5. Conclusions

Among the 10 studies that examined BDNF level outcomes after resistance training, there were large differences between interventions. The main differences were the lengths of the studies (from a single session to 12 weeks total). There were also differences in intensity based on % of 1RM. In five of the studies, only two or three training sessions were conducted, and in five of the studies, significantly more sessions were conducted (fifteen to forty sessions). There are differences between the results obtained with respect to BDNF between these short and longer studies, but it is not possible to draw a conclusion about which interventions (single sessions vs. many sessions) produce a better BDNF response. For this reason, it is safe to say that the results regarding BDNF and the improvements achieved by resistance and strength training are inconclusive. Based on the available studies, it seems that high intensity (70% and more) based on one repetition maximum (1RM) and specific rest periods are required to induce changes in BDNF. Whole-body training or at least lower-body training with free weights and multi-joint movements also seems to produce better results. Martson et al. [28] hypothesize that a large amount of exercise is needed to affect skeletal muscle loading before measuring BDNF in order to peak blood flow levels. And although there are studies that do not support their findings, they believe that this is due to incorrect exercise intensity and excessive rest periods, and the authors of this present systematic review agree with this statement. Goekint et al. [16] is a good example because the intensity seems to be chosen correctly at 80% of 1RM for three sets of 10 repetitions, but they used long recovery periods between sets, which reduced the overall intensity of the training sessions and probably also the exposure to high blood flow in the limbs. Martson et al. [28] also speculate that plasma vs. serum BDNF is the cause of some negative results, such as in Correia et al. [35]. The release of BDNF by platelets is a major source of serum BDNF and is not regulated during high-blood-flow-related shear stress [82]. Therefore, plasma samples measuring peripheral BDNF levels would not capture platelet-realized BDNF, regardless of the exercise intensity.

Further studies are needed to draw a better conclusion for BDNF response to resistance training in healthy young adults.

## Figures and Tables

**Table 1:** Review Summary.

Study	Results	Length	Intensity % of 1RM	Exercise Type	# of Subjects	Age	Gender	Total # of Sessions	Serum or Plasma	Sets x Repetitions	Rest Between Sets
**Lira et al.**	Positive	3 trials	n/a	UB, LB, FB	12	25.3 ± 5.9	men	3	Serum	N/A	N/A
**Church et al.**	Positive	7 weeks	HV-70% HI-90%	MJ, FW	20	23.5 ± 2.6	men	40	Plasma	4 × 10^−12^ 4 × 3^−5^	60 s 180 s
**Yarrow et al.**	Positive	5 weeks	Tradit. 52.5–75% Eccent. 50–120%	n/a	20	college age	men	15	Serum	N/A	60 s
**Marston et al.**	Positive	2 sessions	Maximum weight for 5 and 10 repetitions	FW, FB, MJ, Machines	11 male 5 female	male: 25.0 ± 1.3 female: 23.2 ± 1.1	men and women	2	Serum	3 × 10 5 × 5	60 s 180 s
**Quiles et al.**	Negative	6 weeks	LR–avg. 80% HR–avg. 65%	n/a	15	23 ± 3	men	18	Plasma	8 × 6, 9 × 4, 10 × 2 4 × 12, 4 × 10, 5 × 8	N/A
**Lodo et al.**	Negative	2 weeks (2 trials)	70% 35%	Chest press and squat	30 (15 male and 15 female)	male: 22.8 ± 2.3 female: 22.2 ± 1.7	men and women	2	Serum	4 × 5 4 × 10	120 s
**Vega et al.**	Negative	2 trials	Trial 1–110% Trial 2–40%	KE	11	n/a	n/a	2	Serum	N/A	N/A
**Correia et al.**	Negative	2 trials	60 deg/s	KE, EF	16	26.4 ± 3.7	men	2	Plasma	5 × 10	40 s
**Goekint et al.**	Negative	10 weeks	50–80%	n/a	15	n/a	n/a	n/a	Serum	3 × 10	N/A
**Shiffer et al.**	Negative	12 weeks	70–80%	FB, Machine	27	22.2 ± 1.8	n/a	36	Plasma	3 × 8^−10^	N/A

N/A—not available, HI—high intensity, HV—high volume, 1RM—1 rep maximum, UB—upper body, LB—lower body, FB—full body, LR—lower repetition, HR—higher repetition, Tradit.—traditional training, Eccent.—eccentric enhanced, MJ—multi joint, FW—free weight, BP—bench press, SQ—squat, KE—knee extension, EF—elbow flexion

**Table 2:** Strength adaptation based on tempo.

Time Under Tension	Adaptation
0–20 s	Relative Strength
20–40 s	Functional Hypertrophy
40–70 s	Hypertrophy
70 s or more	Endurance

Own modification based on the original published by Poliquin Principles, 1994 [39].

**Table 3:** 1RM Continuum.

Number of Repetitions	% of Maximum	Training Effect
1	100.0	Relative strength increases through enhanced neural drive
2	94.3	
3	90.6	
4	88.1	
5	85.6	
6	83.1	Optimal compromise of maximal strength and hypertrophy
7	80.7	
8	78.6	
9	76.5	Best hypertrophy gains leading to increased maximal strength
10	74.4	
11	72.3	
12	70.3	
13	68.8	Strength endurance gains and lower hypertrophy gains
14	67.5	
15	66.2	
16	65.0	
17	63.8	
18	62.7	
19	61.6	
20	60.6	

Own modification based on the original published by Poliquin Principles, 1994 [39].

## Data Availability

Not applicable.
